# A Proposed Molecular Mechanism of High-Dose Vitamin D3 Supplementation in Prevention and Treatment of Preeclampsia

**DOI:** 10.3390/ijms160613043

**Published:** 2015-06-09

**Authors:** Piotr Zabul, Michal Wozniak, Andrzej T. Slominski, Krzysztof Preis, Magdalena Gorska, Marek Korozan, Jan Wieruszewski, Michal A. Zmijewski, Ewa Zabul, Robert Tuckey, Alicja Kuban-Jankowska, Wieslawa Mickiewicz, Narcyz Knap

**Affiliations:** 1 Department of Obstetrics & Gynecology, the Sw. Wojciech Specialist Hospital, Independent Public Complex of Integrated Health Care Units in Gdansk, 50 Al. Jana Pawła II St., Gdansk 80-462, Poland; E-Mail: marek@korozan.com; 2 Department of Medical Chemistry, Medical University of Gdansk, 1 Debinki St., Gdansk 80-211, Poland; E-Mails: mwozniak@gumed.edu.pl (M.W.); m.gorska@gumed.edu.pl (M.G.); janwieruszewski@gumed.edu.pl (J.W.); alicjakuban@gumed.edu.pl (A.K.-J.); wmick@gumed.edu.pl (W.M.); 3 Department of Dermatology, University of Alabama at Birmingham, VA Medical Center, Birmingham, AL 35294, USA; E-Mail: aslominski@uabmc.edu; 4 Department of Obstetrics & Gynecology, Medical University of Gdansk, 1A Kliniczna St., Gdansk 80-402, Poland; E-Mail: kpreis@gumed.edu.pl; 5 Department of Histology, Medical University of Gdansk, 1 Debinki St., Gdansk 80-211, Poland; E-Mail: mzmijewski@gumed.edu.pl; 6 Department of Anesthesiology & Intensive Care, Medical University of Gdansk, 1 Debinki St., Gdansk 80-211, Poland; E-Mail: ewa.zabul@gmail.com; 7 School of Chemistry and Biochemistry, the University of Western Australia, 35 Stirling Highway, Crawley, WA 6009, Australia; E-Mail: robert.tuckey@uwa.edu.au

**Keywords:** preeclampsia, isoprostanes, arachidonic acid hydroperoxide, vitamin D3, placenta

## Abstract

A randomized prospective clinical study performed on a group of 74 pregnant women (43 presenting with severe preeclampsia) proved that urinary levels of 15-F_2t_-isoprostane were significantly higher in preeclamptic patients relative to the control (3.05 *vs.* 2.00 ng/mg creatinine). Surprisingly enough, plasma levels of 25-hydroxyvitamin D_3_ in both study groups were below the clinical reference range with no significant difference between the groups. *In vitro* study performed on isolated placental mitochondria and placental cell line showed that suicidal self-oxidation of cytochrome P450scc may lead to structural disintegration of heme, potentially contributing to enhancement of oxidative stress phenomena in the course of preeclampsia. As placental cytochrome P450scc pleiotropic activity is implicated in the metabolism of free radical mediated arachidonic acid derivatives as well as multiple Vitamin D_3_ hydroxylations and progesterone synthesis, we propose that Vitamin D_3_ might act as a competitive inhibitor of placental cytochrome P450scc preventing the production of lipid peroxides or excess progesterone synthesis, both of which may contribute to the etiopathogenesis of preeclampsia. The proposed molecular mechanism is in accord with the preliminary clinical observations on the surprisingly high efficacy of high-dose Vitamin D_3_ supplementation in prevention and treatment of preeclampsia.

## 1. Introduction

A rise in the arterial blood pressure resulting in the development of preeclampsia has been a considerable obstetric problem for years. Eclampsia-related complications can put both the fetus and the mother in jeopardy. The risk of preeclampsia is elevated in the presence of chronic renal disease, chronic hypertension, antiphospholipid syndrome, family history of preeclampsia, multiple pregnancy, maternal age over 40, nulliparity or diabetes [1,2]. The role of oxidative stress in the eclampsia-related complications remains controversial. For many years, there has been no substantial change in the management of preeclampsia and eclampsia. It is not uncommon that when medication fails to normalize the blood pressure, the physician is forced to terminate pregnancy in order to save the mother’s life at the expense of the baby.

In light of numerous clinical studies and literature data, pathogenesis of preeclampsia seems to be complex and depends on multifactorial abnormalities observed at the molecular level [2]. The majority of experts in the field associate etiology of preeclampsia with endothelial injury, oxidative stress phenomena, compromised placental perfusion, imbalance between prostacyclin and thromboxane signaling, decreased glomerular filtration rate with retention of salt and water, alteration of gene expression, and dietary factors including vitamin deficiency [3,4]. Reactive oxygen species (ROS) affect vascular reactivity through multiple mechanisms [5]. Interestingly*,* Mousa *et al.* [6] reported that reduced methylation of the thromboxane synthase gene correlated with its increased vascular expression in preeclampsia. Oxidative stress, as measured by placental isoprostane generation [7], caused DNA hypomethylation, and preeclampsia was associated with oxidative stress [6]. Interestingly, 15-F_2t_-isoprostane (15-F_2t_-isoP), a product of nonenzymatic lipid peroxidation, is a potent renal vasoconstrictor acting principally through thromboxane A2 receptor activation [8], and thus enhances the biological effects of thromboxane synthase gene expression. In addition, 15-F_2t_-isoP has been implicated as a causative mediator in hepatorenal syndrome [9]. There are clinical trials suggesting that pharmacological lowering of 15-F_2t_-isoP urinary excretion might improve an antihypertensive treatment in patients presenting with chronic kidney failure [10,11].

Vitamin D_3_ is naturally produced in the skin subjected to ultraviolet radiation from sun light. It is estimated that approximately 80%–100% of daily Vitamin D_3_ supply comes from synthesis in the skin, with only limited contribution from dietary intake [12]. Vitamin D_3_ deficiency in early pregnancy can constitute an independent causal factor for the development of eclampsia [13] since high incidence rates are reported to coincide with the winter months [14], when cutaneous synthesis of Vitamin D_3_ is lacking due to the absence of UVB in solar light in this geographical area [12].

The activation of Vitamin D_3_ requires its subsequent enzymatic hydroxylations at carbons C25 and C1. Initial hydroxylation at C25 in the liver produces 25(OH)D_3_ which due to its relatively longhalf-life of 2–3 weeks in the human body, is routinely used as an indicator of the overall Vitamin D status [15]. The second hydroxylation producing the hormonally active 1α,25-dihydroxyvitamin D_3_ (calcitriol, 1α,25(OH)_2_D_3_) takes place in the kidney. Active metabolites of Vitamin D display an array of biological effects mediated through genomic and non-genomic pathways [16,17]. In many cells and tissues, 1α,25(OH)_2_D_3_ first binds to the Vitamin D receptor (VDR) which then forms a heterodimer with the receptor for 9-cis retinoic acid (RXR); this dimer is translocated to the nucleus where it controls expression of over 1000 genes [18]. Calcitriol (1,25(OH)_2_D_3_), an active form of Vitamin D_3_, can serve as a placental vasculature modulator possibly through its antioxidative activities [19,20]. The endogenous formation of Vitamin D exclusively depends on sun exposure (UVB wavelengths 280–320 nm) [12], and many studies support seasonal variation in the prevalence of preeclampsia [21]. There is evidence that oral supplementation of Vitamin D can decrease the rate of severe preeclamptic complications [22,23].

An imbalance between oxidant and antioxidant potential is associated with reproductive problems [24]. The level of lipid peroxide in the maternal blood is significantly elevated in preeclampsia as compared to normal pregnancy, and levels of antioxidants are compromised [25,26,27,28]. However, some authors report that there are no differences in 15-F_2t_-isoP, lipid peroxide or malondialdehyde plasma levels between women with preeclampsia and pregnant controls [29]. Importantly, the placenta produces large amounts of progesterone required for the maintenance of pregnancy, synthesizing 10 times the quantity secreted by the corpus luteum in the mid-luteal phase [30]. It is well known that placental mitochondria are responsible for progesterone biosynthesis [31] and preeclampsia may develop because of an inadequate supply of progesterone [32]. It is likely that in the preeclamptic placenta progesterone synthesis may be compromised [33]. Conversion of cholesterol to progesterone starts with the production of pregnenolone, catalyzed by cytochrome P450scc (side-chain cleavage cytochrome P450; CYP11A1) in the inner mitochondrial membrane [30,31,34]. In the placenta cytochrome P450scc also hydroxylates Vitamin D_3_ producing 20-hydroxyvitamin D_3_ (20(OH)D_3_) which can be further transformed to other biologically active metabolites [35,36]. The existence of gene transcript of 1α-hydroxylase and the finding of Vitamin D receptor (VDR) expression in placental trophoblasts suggest a possible autocrine loop of Vitamin D signaling within trophoblasts [37,38]. The unique metabolic profile of the placental cytochrome P450scc appears to arise from its location in trophoblast cells, as compared to the adrenal cortex and gonads [30], as well as from the local exposure to a broad range of substrates produced during pregnancy. Among arachidonic acid metabolites, isoprostanes are unique bioactive (vasoconstrictive) products of cytochrome P450scc-mediated lipid peroxidation. The synthetic hydroperoxide, cumene hydroperoxide, has been found to inactivate cytochrome P450scc in human term placental mitochondria [32].

An objective of this study was to evaluate Vitamin D_3_ and oxidative stress levels in a population of pregnant women with severe preeclampsia/eclampsia in order to verify potential correlation between wintertime plasma levels of 25-hydroxyvitamin D_3_ (25(OH)D_3_) as measured in late gestation, and urinary excretion of 15-F_2t_-isoprostane which is a reliable marker of oxidative stress in the human body. A prospective study was designed in order to measure urinary levels of isoprostanes as markers of oxidative stress and potential vasoconstrictors, and levels of Vitamin 25(OH)D_3_ whose deficiency might be a potential cofactor for the development of eclampsia. The study comprised caucasian women of northern Poland who were admitted to the hospital from December through February, and subsequently qualified for C-section.

As placental cytochrome P450scc is required for progesterone synthesis, oxidative stress generation and metabolism of Vitamin D_3_, a parallel study on the activity of placental cytochrome P450scc under oxidative stress conditions, as well as lipid peroxidation in placental cells, was carried out. To determine whether increased levels of naturally synthesized arachidonic acid hydroperoxide had a similar impact on lipid peroxidation and biodegradation of cytochrome P450scc, we measured progesterone synthesis by isolated mitochondria from term human placentas, and measured lipid peroxidation by placental mitochondria and cultured placental JAR cells.

## 2. Results

**Figure 1:**
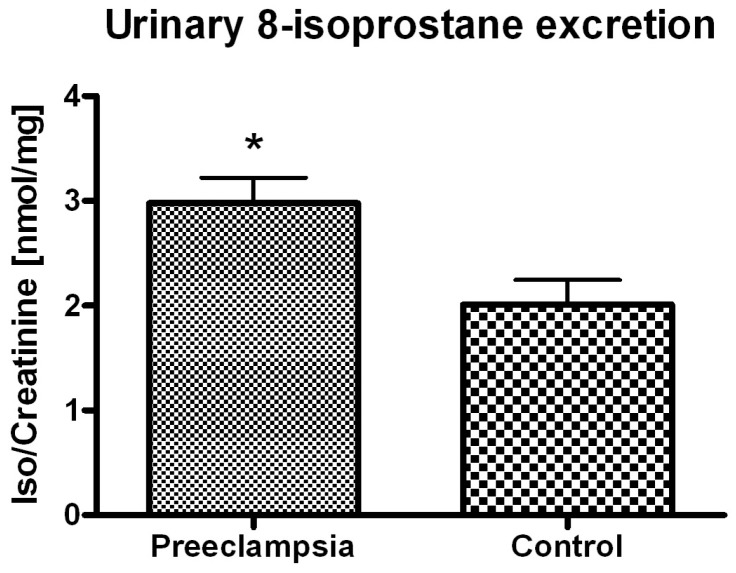
Urinary isoprostane excretion as calculated per mg of creatinine in preeclamptic women *vs.* control (* *p* < 0.01).

**Figure 2:**
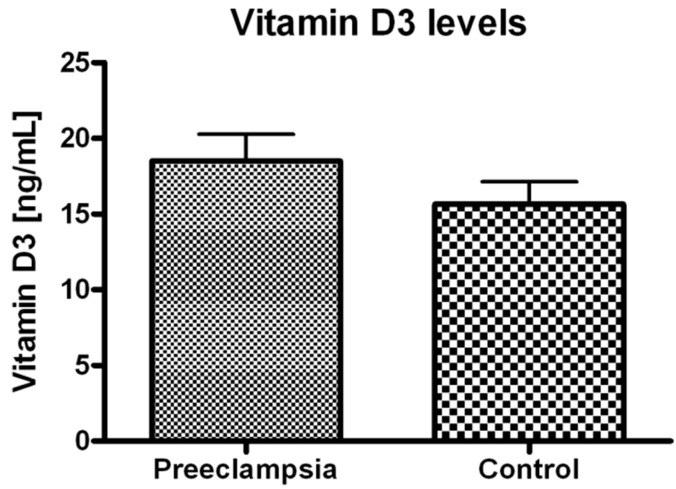
Vitamin D_3_ (25(OH)D_3_) plasma levels in preeclamptic women *vs.* control. No significant difference between groups was found.

The urinary levels of 15-F_2t_-isoP in the preeclamptic group were significantly higher relative to the control group (3.05 *vs.* 2.00 ng/mg creatinine; *p* < 0.01) (Figure 1). This finding supports the hypothesis that biological effects of an oxidative stress-mediated increases in placental generation of thromboxane A2 may be enhanced by 15-F_2t_-isoP isoprostane-dependent activation of the thromboxane A2 receptor [8]. The plasma levels of 25(OH)D_3_ in both groups were below the clinical reference range of 30 ng/mL with no difference between the groups (mean 16.8 ng/mL) (Figure 2). There was no correlation between the urinary excretion of 15-F_2t_-isoP and plasma levels of 25(OH)D_3_ in either of the study groups.

**Figure 3:**
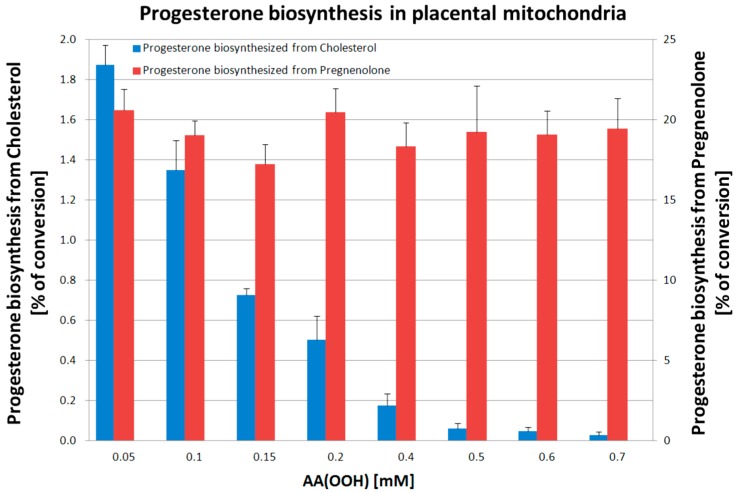
Progesterone biosynthesis as a function of AA(OOH) concentration in placental mitochondria (measured as percentage of substrate conversion). Progesterone biosynthesis from cholesterol or pregnenolone was measured following a 15 min. incubation. Data presented as mean ± SD obtained from five independent experiments.

**Figure 4:**
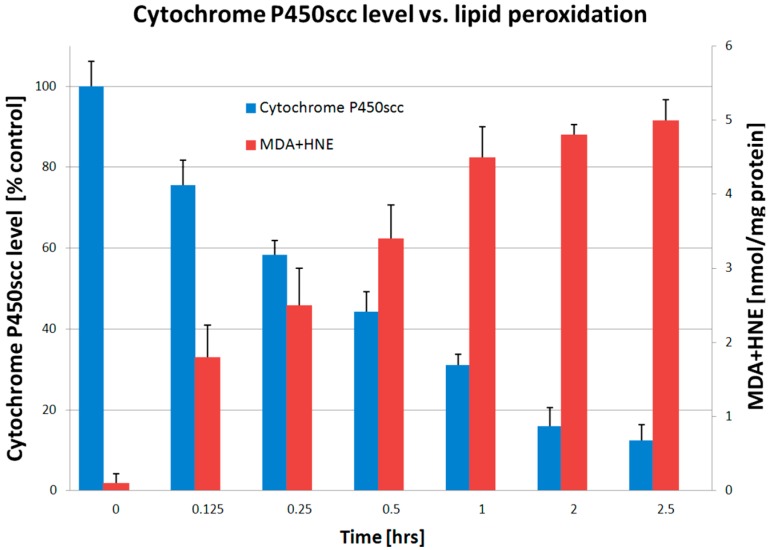
Cytochrome P450scc level after treatment with arachidonic acid hydroperoxide (AA(OOH)). Lipid peroxidation induced by AA(OOH) was measured from the production of MDA and HNE. Data are presented as mean ± SD obtained from five independent experiments expressed as percentage of control.

To test the effect of a natural lipid peroxide on progesterone production, increasing concentrations of AA(OOH) were added to placental mitochondria (Figure 3). This resulted in the inhibition of progesterone synthesis from cholesterol but no effect on its synthesis from pregnenolone. This result indicates that P450scc required to convert cholesterol to pregnenolone, and not 3β-hydroxysteroid dehydrogenase required to convert pregnenolone to progesterone, is the locus of action of the AA(OOH). To determine whether the decrease in P450scc activity was associated with a decrease in P450scc levels, the concentration of functional P450, assessed from its ability to bind CO, was measured following treatment of mitochondria with 100 µM AA(OOH). The cytochrome P450 concentration decreased in a time-dependent manner which was inversely proportional to the increase in the concentration of lipid peroxidation products MDA and HNE (Figure 4). This indicates that inactivation/degradation of P450scc occurs in response to AA(OOH) treatment, possibly as a result of heme breakdown [32,39,40,41,42,43,44].

**Figure 5:**
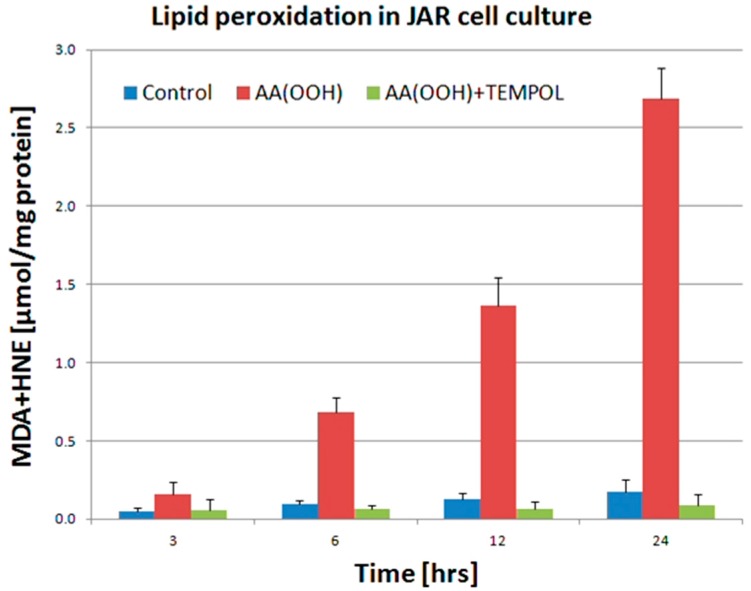
Lipid peroxidation in JAR cell culture. Control cells, cells treated with 100 µM Arachidonic Acid Hydroperoxide AA(OOH), cells treated with 100 µM AA(OOH) and 50 µM TEMPOL (AA(OOH) + TEMPOL). Products of lipid peroxidation (MDA plus HNE) were significantly elevated in cells treated with AA(OOH) only. Data presented as mean ± SD obtained from five independent experiments.

To test the ability of AA(OOH) to stimulate lipid peroxidation, the JAR placental cell line was treated with AA(OOH) and products of lipid peroxidation measured. This treatment dramatically stimulated formation of lipid peroxidation products over a 24 h period. Treatment with the powerful antioxidant, TEMPOL, completely protected the cells against the AA(OOH)-induced lipid peroxidation (Figure 5).

## 3. Discussion

The results of our study showed that severe cases of preeclampsia (terminated by C-section) were associated with increased oxidative stress as measured by urinary isoprostane level. Importantly, we chose C-section terminated normal pregnancies as the control group in order to eliminate a potential ROS increase in response to surgical trauma [45]. Our results are in accord with a couple of studies in terms of urinary isoprostane excretion [46,47], and contradict a few others [48,49,50]. There has been a lot of controversy about circulating oxidative stress markers in preeclampsia, and specifically isoprostanes as measured in different compartments [51,52]. Isoprostanes, and typically 15-F_2t_-isoP as evaluated in plasma or urine, have been applied in clinical studies as reliable markers of total body oxidative stress [53]. A decreased urinary excretion of 15-F_2t_-isoP is attributed to reactive oxygen species scavenging activity of certain antihypertensive drugs used for therapy in chronic renal failure therapy [10,11]. Walsh *et al.* [7] provided convincing evidence that F(2)-isoprostanes are formed and secreted by the human placenta, and that oxidative stress and lipid peroxidation are pathologically increased in placentas of preeclamptic women . It is only logical that measurement of isoprostanes in body fluids could offer a unique analytical opportunity to study the role of free radicals in pathophysiology of preeclampsia. There are however many contradictory observations in the literature in terms of isoprostane levels in the plasma or urine of preeclamptic women. According to several studies, there was a significant increase observed in total or free isoprostane plasma levels in preeclampsia [48,49,54]. Other researchers did not report any difference in isoprostane plasma levels between normal pregnancy and preeclampsia [29,55], including severe preeclampsia [50]. The difference in the levels of isoprostanes in various studies might possibly be due to methodology (ELISA (Enzyme-linked Immunosorbent Assay), RIA (Radioimmunoassay), GC (Gas Chromatography)), different sampling regime, gestational age, social profile, geographical area, and ethnicity to name but a few. Taking into account that renal clearance of isoprostane may be impaired in preeclampsia, urinary isoprostane level should be preferably corrected for creatinine [47,54] which has not always been the case [48,49]. In light of multiple conflicting data, our finding of increased urinary isoprostane excretion in severe preeclampsia adds to existing evidence for a role of oxidative stress in pathogenesis of the disease which seems all the more significant as there are researchers who tend to question the importance of oxidative stress phenomena as the biochemical rationale for etiopathology, prevention and treatment of preeclampsia [55,56].

Pleiotropic effects of Vitamin D_3_ have been the focus of many publications, linking Vitamin D_3_ with placental regulation and modulation of inflammatory response [57,58]. Also, clinical studies show higher rates of preeclampsia, preterm birth, bacterial vaginosis and gestational diabetes in women with low Vitamin D levels [59]. A large population of pregnant women is affected by significant Vitamin D_3_ deficiency [60,61,62]. Indeed, our results confirm that Vitamin D_3_ deficiency might be a more general clinical problem in the northern European population of pregnant Caucasian women suffering from limited sun exposure during the winter season [63,64]. Interestingly, a recent autumn–winter study on the urban population of northern Poland showed that 85% of participants was Vitamin D deficient, with mean 25-hydroxyvitamin D_3_ concentration of 14.3 ± 6.6 ng/mL [65]. Furthermore, the subsequent study showed only moderate improvement of Vitamin D_3_ status after the summer [66]. It should be noted that in our current study, the mean plasma level of Vitamin D_3_ as calculated for both preeclamptic and control groups was 16.8 ng/mL and the difference between the groups was not statistically significant. Thus, a lack of correlation between 15-F2t-isoP and 25(OH)D_3_ might be partly explained by the substantial Vitamin D_3_ deficiency in both study groups. These observations should encourage obstetricians and gynecologists to reconsider additional Vitamin D_3_ supplementation in pregnant women, and in particular those who belong to the risk group being potentially hypersensitive to Vitamin D_3_ deficiency [13,67,68].

As mitochondria reportedly contribute to increased lipid peroxidation in the preeclamptic placenta [69,70], we decided to design a mitochondrial model of preeclampsia based on placental mitochondria isolated from healthy women, and treated with AA(OOH) in order to induce well characterized oxidative stress phenomena as observed in the preeclamptic trophoblast cells [70,71,72]. The *in vitro* model was then used in our study to evaluate the enzymatic activity of cytochrome P450scc in response to AA(OOH)-mediated oxidative stress. Cytochrome P450scc is a key mitochondrial enzyme in steroid hormone synthesis, lipid peroxidation and trophoblastic cell behavior, which is integral to the pathogenesis of preeclampsia [73]. The expression of *CYP11A* gene encoding P450scc protein was reported to be significantly higher in terms of both mRNA and protein levels, and was proposed to be linked with abnormal apoptosis of trophoblastic cells in placentas of preeclamptic patients [73,74]. Having observed similar mitochondrial degeneration followed by massive apoptosis in choriocarcinoma JAR cell line exposed to oxidative stress [75,76], we ventured to propose a novel mitochondrial model of preeclampsia allowing for *in vitro* characterization of cytochrome P450scc activity and structural integrity under the conditions of AA(OOH)-induced oxidative stress. The effect of oxidative stress on reducing the activity of cytochrome P450scc seen in the current study suggests that an impairment in the production of P450scc-derived Vitamin D_3_ metabolites may be expected. There is evidence of disrupted Vitamin D metabolic homeostasis in the preeclamptic placenta suggesting that increased oxidative stress could be a causative factor of altered Vitamin D metabolism therein [77,78]. Although preeclampsia has been linked to maternal Vitamin D insufficiency [13,62,78,79], the information on placental Vitamin D metabolic system between normal and preeclamptic pregnancies is lacking [77,80]. It is entirely possible that it is not the plasma concentration of Vitamin D_3_, but rather the local placental concentration of Vitamin D_3_ or its metabolites in combination with oxidative stress-generated arachidonic acid derivatives that might actually play a role in the prevention or treatment of preeclampsia/eclampsia.

Our *in vitro* experiments demonstrate that reduced cytochrome P450scc activity upon increased preeclamptic flux of AA(OOH) (Figure 3) is associated with heme disintegration (Figure 4), and consequently the release of free iron Fe^2+^ ion, analogously to what has been reported for hemoglobin [81], which in turn induces Fenton-like reactions enhancing lipid peroxidation [81,82] as measured by increasing MDA and HNE levels (Figure 4). Under *in vivo* conditions, there are many other lipid peroxidation products, like lipid peroxides [70,71,83,84,85], hydroperoxides [86], alkyl radicals [87], alkoxyl radicals [86] or F_2_-isoprostanes [7,8,48,49,54] that are generated in excess and typically detected in the placenta and plasma of preeclamptic patients contributing to increased vasoconstriction and development of arterial hypertension. Surprisingly enough, preeclampsia is rarely associated with decreased P450scc activity and inadequate synthesis of placental progesterone [88]. On the contrary, most studies report significant increase in placental progesterone production in the course of preeclampsia [83,89] linking progesterone directly with an imbalance between prostacyclin PGI_2_ and thromboxane A_2_ production in favor of the latter [84,90,91,92]. Significant DNA hypomethylation was observed in preeclampsia for steroidogenic genes, including *CYP11A1* for cytochrome P450scc and *HSD3B1* for 3β-hydroxy-delta-5-steroid dehydrogenase type 1, each controlling the two-step pathway of progesterone synthesis from cholesterol [93]. As DNA methylation is inversely associated with mRNA expression, both transcripts were accordingly elevated in patients presenting with either early or late onset preeclampsia compared to controls. CYP11A expression was significantly increased in severe preeclampsia compared with normal pregnancy in both mRNA and protein levels [73,74]. Maternal progesterone levels as measured either in the placenta or plasma were increased in women with preeclampsia [89,94], and both progesterone and estradiol were reported to positively stimulate *CYP11A1* and *HSD3B1* expression in trophoblast cells increasing the abundance of P450scc and 3β-HSD type 1 mRNAs but had no significant effect on the amount of 3β-HSD protein [95]. Circulating or urinary concentrations of progesterone were reported to be either within the normal range [83,96] or higher [74,94] in preeclamptic women. Therefore, it seems justified to presume that pathologically elevated progesterone may act in a compensatory feedforward loop to excessively promote placental steroidogenesis [93,95], and thus propel a metabolic vicious circle increasing progesterone level and progesterone-dependent vasoconstriction as observed in preeclampsia [71,74,89,90,91,94]. Moon *et al.* [74] have recently presented a very convincing study based on GC-MS (Gas Chromatography-Mass Spectrometry) metabolic profiling where plasma pregnenolone and progesterone were significantly increased (>2.0-fold, *p* < 0.001) in preeclamptic patients as compared with control subjects, while cholesterol was significantly decreased (<1.4-fold, *p* < 0.001). Progesterone is essential for the maintenance of human pregnancy. However, elevated progesterone concentrations could on the one hand suppress the production of the potent vasodilator, prostacyclin, and on the other, stimulate the synthesis of the potent vasoconstrictor, thromboxane [71,74,89,90,91]. In light of the above analysis, progesterone should not be advocated for prevention of preeclampsia and its complications [97] as it used to be in the past.

There is a growing number of reports linking maternal Vitamin D_3_ deficiency with preeclampsia [13,68,78,79,80,98]. The *in vivo* studies represented by *ex-utero* incubations of Vitamin D_3_ with fragments of human placentas demonstrated the CYP11A1-catalyzed hydroxylation of Vitamin D_3_ to 20(OH)D_3_ being the major metabolite [99]. It is important to note that human CYP11A1 does not metabolize 25(OH)D_3_ [100], and does not interfere with placental activation of 25-hydroxyvitamin D_3_ [101]. Therefore, it is Vitamin D_3_ rather than 25(OH)D_3_ possibly competing with cholesterol for the catalytic center of P450scc. Taking into account that the Km needed for the conversion of Vitamin D_3_ to 20(OH)D_3_ by placental CYP11A1 is higher than that for the metabolism of cholesterol [102], only a relatively high level of Vitamin D_3_ will competitively inhibit excess pregnenolone synthesis, and restore local placental production of 20(OH)D_3_ and other derivatives by P450scc under the metabolic conditions of preeclampsia. As a matter of fact, our preliminary clinical observations suggest a plausible therapeutic effect of high-dose oral supplementation with 4000 IU Vitamin D_3_ on preeclamptic placental vasculature which might be related to the competitive inhibition of abnormal pregnenolone and subsequent progesterone synthesis, as well as integral stabilization of placental cytochrome P450scc activity, and consequent reduction in lipid peroxidation. In our material on hypertensive patients, not only did high-dose Vitamin D_3_ supplementation normalize arterial blood pressure, it also improved uterine vasculature as confirmed by ultrasound (data not shown) which accords with the Norwegian Mother and Child Cohort Study [103] and other correlational studies [13,68,80].

According to the WHO “Guideline for vitamin D supplementation in pregnant women” (Geneva, World Health Organization, 2012), Vitamin D_3_ supplementation is not recommended during pregnancy to prevent the development of preeclampsia and its complications unless in cases of documented deficiency where Vitamin D_3_ supplements may be given at the current RNI (recommended nutrient intake) of 5 μg (200 IU) per day as recommended by WHO/FAO or according to national guidelines. However, supplementation has been shown to have minimal toxicity in adults receiving doses of up to 10,000 IU per day [104,105]. Vitamin D toxicity generally becomes evident at doses of 20,000 IU per day and can lead to hypercalcaemia, hypercalciuria, and elevated (200 nmol/L) levels of serum 25(OH)D [106]. There are few safety studies in pregnant women, however, in one recent study, up to 4000 IU Vitamin D_3_ was provided to pregnant women from the twelfth to sixteenth weeks of pregnancy until delivery, with no reported cases of hypercalcaemia or hypercalciuria [107]. Current evidence supports the concept that circulating 25-hydroxyvitamin D during pregnancy should rather be 40–60 ng/mL (100–150 nmol/L) suggesting either a high-dose daily intake of 4000 IU Vitamin D_3_ or high-dosage interval bolus (35,000 IU/week or more) in order to attain that circulating level [60,108,109]. Importantly, according to a couple of animal studies, 20(OH)D_3_, being the major placental metabolite of Vitamin D_3_, was reported to show a lack of calcemic or other toxic effects (as determined by serum chemistry and histological analyses of heart, spleen, liver, and kidney) at pharmacological doses far above the toxicity levels of 25(OH)D_3_ or 1,25(OH)_2_D_3_ [110,111,112]. Moreover, 20(OH)D_3_ exhibited potent anti-inflammatory [113,114], antifibrogenic [112] and anticancer [110,115] properties, all of which being potentially beneficial in the prevention or therapy of preeclampsia.

**Figure 6:**
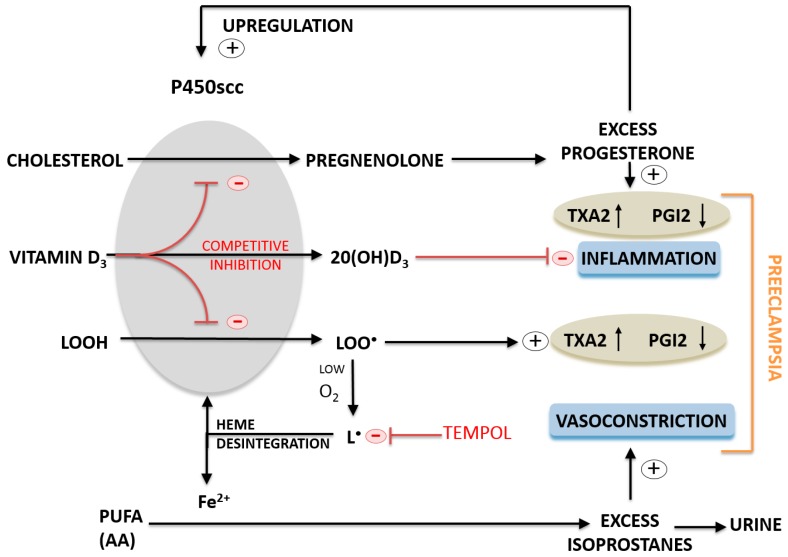
A proposed molecular mechanism of high-dose Vitamin D_3_ supplementation in prevention or treatment of preeclampsia. Vitamin D_3_ acts as a competitive inhibitor of placental cytochrome P450scc preventing the production of lipid peroxides and excess progesterone, both of which may contribute to the etiopathogenesis of preeclampsia. 4-OH-TEMPO (TEMPOL) protects placental mitochondria as an effective scavenger of carbon-centered radicals.

We proposed a cellular model of preeclampsia exposing JAR trophoblast cells to AA(OOH)-induced lipid peroxidation analogously to our mitochondrial experimental model of preeclampsia which has been discussed above. Biochemical and ultrastructural changes as observed in JAR cell line exposed to oxidative stress phenomena had been previously defined [75,76], and were in accord with the characteristics of preeclamptic placental cells [116]. We observed the same classical products of lipid peroxidation, namely MDA and HNE as we did in the isolated placental mitochondria having been exposed to AA(OOH). Lipid peroxidation was completely inhibited in cell culture pretreated with 50 µM TEMPOL (AA(OOH)+TEMPOL). Interestingly enough, contrary to common enzymatic antioxidants, nitroxides like 4-OH-TEMPO (TEMPOL) can provide protection of biological systems from oxidative stress by pre-emptying of carbon-centered radicals in lipid peroxidation chain reaction [117,118], thus preventing cytochrome P450scc heme destruction, which might be the mechanism of the observed reduction in P450scc activity (Figure 3) and concentration (Figure 4). High protective efficacy of 4-OH-TEMPO in the placental cell culture model (Figure 5 and Figure 6) can be explained by extremely high absolute rate constant for the cross-coupling reaction of several carbon-centered radicals with various nitroxides (2.3 × 10^9^ M^−1^·s^−1^) [117,119,120]. As opposed to 4-OH-TEMPO, Vitamin E preferentially scavenges peroxyl radical, and proved to be ineffective in the prevention of preeclampsia [56,121]. The results of this study allow us to propose that alkyl radicals rather than peroxyl radicals are mainly involved in the oxidative damage to trophoblast cells under the conditions of preeclampsia (Figure 6).

In summary, our results suggest that regardless of inconsistencies found in literature, monitoring isoprostane concentration in the urine of pregnant women could be a valuable noninvasive method of measuring oxidative stress and it could also serve as an indicator for the initiation of anti-inflammatory therapy, or high-dose Vitamin D_3_ supplementation throughout gestation. We propose that Vitamin D_3_ might act as a competitive inhibitor of placental cytochrome P450scc preventing from the production of lipid peroxides or excess progesterone synthesis, both of which may contribute to the etiopathogenesis of preeclampsia.

## 4. Experimental Section

### 4.1. Clinical Study Design

The investigational group comprised patients with direct indications for having the pregnancy terminated by caesarian section, *i.e.*, preeclampsia due to uncontrolled rise in blood pressure in spite of antihypertensive treatment. The control group was made up of pregnant patients with other obstetric indications for C-section, such as breech presentation, cephalopelvic disproportion or dystocia. All of the patients consented to participate in the study. The study was continued for two winter seasons; concomitant medical conditions in the mother and low birth weight in the newborn were considered exclusion criteria. In the end, the preeclamptic cohort included 43 women with severe preeclampsia (clinical profile presented in Table 1). There were 31 women with no symptoms of preeclampsia included as the control. Sociodemographic characteristics of the two study groups were comparable (Table 1). Study samples were obtained from urine collected for approximately 24 h, from the time when urinary catheterization was performed as the patient was qualified for C-section, up until the last post-op fluid administration. Urine samples were taken from the drainage bag and stored at −80 °C. 25(OH)D_3_ levels were determined on plasma samples taken immediately after caesarian section, as part of the routine blood count and electrolyte panel. Both plasma and urine were aliquoted, and stored at −80 °C for further immunochemical analysis.

**Table 1:** Sociodemographic and clinical profiles of the preeclamptic *vs.* non-preeclamptic cohort.

Characteristic	Non-Preeclamptic (Mean ± SD)	Preeclamptic (Mean ± SD)	*t*-Test (*p*-Value)
Age (years)	29 ± 6	30 ± 7	*p* > 0.3
Systolic blood pressure (mm Hg)	122 ± 8	164 ± 7	*p* < 0.0001
Diastolic blood pressure (mm Hg)	74 ± 10	99 ± 5	*p* < 0.0001
Gravidity	1.7 ± 1.0	1.6 ± 1.1	*p* > 0.2
Parity	0.5 ± 0.7	0.4 ± 0.6	*p* > 0.1
Gestational age (weeks)	39 ± 2	37 ± 3	*p* < 0.001
Newborn’s weight (g)	3500 ± 500	2780 ± 900	*p* < 0.0001
Ethnicity	Caucasian	Caucasian	NA

### 4.2. Immunochemical Assays

A commercial ELISA kit (Cayman Chemical Co., Ann Arbor, MI, USA) was used to measure the urinary excretion of 15-F_2t_-isoprostane (15-F_2t_-isoP), widely accepted as a sensitive marker of oxidative stress in the human body [53]. The level of 15-F_2t_-isoP as immunochemically assessed in the urine was then calculated relative to urinary creatinine content and expressed as ng/mg of creatinine. 25(OH)D_3_ was measured in plasma samples following a routine procedure applied by the hospital laboratory (Elecsys Vitamin D assay; normal values were ≥30 ng/mL). 4-hydroxynonenal (HNE) was assessed using OxiSelect HNE Adduct ELISA Kit (Cell Biolabs, Inc., San Diego, CA, USA).

### 4.3. Preparation of Placental Mitochondria

Human term placental mitochondria were prepared as described previously [122].

### 4.4. Cell Culture

The choriocarcinoma cell line JAR (ATCC HTB-144) was cultured in a humidified atmosphere with 5% CO_2_ in RMPI-1640 (Sigma-Aldrich Co., St. Louis, MO, USA) containing 1 mM sodium pyruvate, 10 mM Hepes (Sigma-Aldrich, Poznan, Poland), supplemented with 10% heat-inactivated fetal bovine serum (Gibco, Grand Island, NY, USA), penicillin (100 IU/mL) and streptomycin (100 µg/mL) (Sigma-Aldrich, Poznan, Poland).

### 4.5. Cell Treatment with an Oxidative Stress Inducer

Cells cultured on 6-well culture dishes (5 × 10^5^–1 × 10^6^ cells/well) were treated for 3, 6, 12 or 24 h with 100 µM arachidonic acid hydroperoxide: 15(s)hydroperoxy(5*Z*,8*Z*,11*Z*,13*E*)-eicosatetraenoic acid (AA(OOH)) (Sigma-Aldrich, Poznan, Poland) in the culture medium. The cells of the TEMPOL-pretreated group were incubated for 2 h with 50 µM TEMPOL (4-hydroxy-TEMPO) (Sigma-Aldrich, Poznan, Poland) in the culture media prior to addition of 100 µM AA(OOH). Control cells did not receive any treatment. Cells (5 × 10^6^ cells/mL) were collected from the culture dishes by trypsinization, centrifuged together with those floating in the culture medium and were washed with PBS. The cells were then lysed with lysis buffer and subjected to analysis of lipid peroxidation products as described below. Protein was determined using Bradford method (1976) after dissolving the perchloric acid precipitates in 0.5 M NaOH.

### 4.6. Placental Mitochondria Treatment with Oxidative Stress Inducer

A suspension of placental mitochondria was incubated with 100 µM arachidonic acid hydroperoxide (AA(OOH)) for 0, 0.125, 0.25, 0.5, 1.0, 2.0 and 2.5 h at 37 °C under air with constant shaking in 2.5 mL medium containing 0.1 M Tris-HCl buffer (pH 7.4), and 5 mg of mitochondrial protein. The levels of mitochondrial cytochrome P450 was determined as percentage of the control by the method of Omura and Sato [123], using a molar absorption coefficient 100,000 M^−1^·cm^−1^ for the difference in absorbance between 450 and 490 nm [124].

Lipid peroxidation was assessed using commercial kits as described below.

### 4.7. Lipid Peroxidation Assays

Lipid peroxidation in cells lysates or placental mitochondria suspensions were evaluated on the basis of increasing levels of the key lipid peroxidation products malondialdehyde (MDA) and 4-hydroxynonenal (HNE). MDA was determined by a colorimetric method using a Lipid Peroxidation (MDA) Assay Kit (Abcam, Cambridge, UK) according to manufacturer’s instruction. HNE was determined immunochemically using an OxiSelect HNE Adduct ELISA Kit (Cell Biolabs, Inc., San Diego, CA, USA) according to the manufacturer’s instruction.

### 4.8. Progesterone Biosynthesis 

NADP^+^, glucose-6-phosphate, glucose-6-phosphate dehydrogenase were obtained from Sigma-Aldrich, Poland. [4-^14^C]Cholesterol (58 mCi/mmol), [4-^14^C]pregnenolone (55 mCi/mmol), [^3^H]progesterone (12 Ci/mmol) and [^3^H]pregnenolone (6.9 Ci/mmol) were obtained from Radiochemical Centre (Amersham, UK). All other materials were of the highest analytical grade available from Sigma-Aldrich (Poland). Progesterone synthesis by mitochondria was measured using an NADPH-generating system consisting of 0.5 mM NADP^+^, 3 mM glucose-6-phosphate and 5 U/mL glucose-6-phosphate dehydrogenase, using radiolabeled precursors as previously described [32].

### 4.9. Statistical Analysis

The results were expressed as mean ± SEM (unless stated otherwise), and the significance of the difference between the mean values relative to control was determined by the Student’s *t* test. Significance was determined at the 5% level (* *p* < 0.05), two-sided. Statistical significance between treatment and control group was indicated by asterisk. Correlation between variables was assessed by Pearson’s r correlation test. Significance of correlation was determined at the 5% level (* *p* < 0.05). All of the statistical calculations were performed using GraphPad Prism 4 software by GraphPad Software, Inc. (La Jolla, CA, USA).

## 5. Conclusions

The study showed a significantly increased level of oxidative stress, as measured by the urinary isoprostane excretion, in women presenting with severe preeclampsia as compared to control. Both study groups were Vitamin D_3_ deficient. There was no correlation between wintertime 25(OH)D_3_ and severe preeclampsia. However, low levels of Vitamin D_3_ metabolites might potentially impair placental cytochrome P450scc activity inducing oxidative stress phenomena and increase the risk of preeclampsia. High-dose supplementation with Vitamin D_3_ seems to be a rational, safe and effective countermeasure. Further randomized trials with supplementation of Vitamin D_3_ or alternative combinations of clinically effective antioxidants in prevention and treatment of preeclampsia are necessary.

## References

[1] Li D.K., Wi S. (2000). Changing paternity and the risk of preeclampsia/eclampsia in the subsequent pregnancy. Am. J. Epidemiol..

[2] Reslan O.M., Khalil R.A. (2010). Molecular and vascular targets in the pathogenesis and management of the hypertension associated with preeclampsia. Cardiovasc. Hematol. Agents Med. Chem..

[3] Li Y.C., Kong J., Wei M., Chen Z.F., Liu S.Q., Cao L.P. (2002). 1,25-Dihydroxyvitamin D_3_ is a negative endocrine regulator of the renin-angiotensin system. J. Clin. Investig..

[4] Holmes V.A., McCance D.R. (2005). Could antioxidant supplementation prevent pre-eclampsia?. Proc. Nutr. Soc..

[5] Takagi Y., Nikaido T., Toki T., Kita N., Kanai M., Ashida T., Ohira S., Konishi I. (2004). Levels of oxidative stress and redox-related molecules in the placenta in preeclampsia and fetal growth restriction. Virchows Arch..

[6] Mousa A.A., Strauss J.F., Walsh S.W. (2012). Reduced methylation of the thromboxane synthase gene is correlated with its increased vascular expression in preeclampsia. Hypertension.

[7] Walsh S.W., Vaughan J.E., Wang Y., Roberts L.J. (2000). Placental isoprostane is significantly increased in preeclampsia. FASEB J..

[8] Takahashi K., Nammour T.M., Fukunaga M., Ebert J., Morrow J.D., Roberts L.J., Hoover R.L., Badr K.F. (1992). Glomerular actions of a free radical-generated novel prostaglandin, 8-epi-prostaglandin F2 α, in the rat. Evidence for interaction with thromboxane A2 receptors. J. Clin. Investig..

[9] Morrow J.D., Moore K.P., Awad J.A., Ravenscraft M.D., Marini G., Badr K.F., Williams R., Roberts L.J. (1993). Marked overproduction of non-cyclooxygenase derived prostanoids (F2-isoprostanes) in the hepatorenal syndrome. J. Lipid Mediat..

[10] Renke M., Tylicki L., Knap N., Rutkowski P., Neuwelt A., Larczynski W., Wozniak M., Rutkowski B. (2008). Spironolactone attenuates oxidative stress in patients with chronic kidney disease. Hypertension.

[11] Renke M., Tylicki L., Knap N., Rutkowski P., Neuwelt A., Petranyuk A., Larczynski W., Wozniak M., Rutkowski B. (2009). High-dose angiotensin-converting enzyme inhibitor attenuates oxidative stress in patients with chronic kidney disease. Nephrol. Dial. Transplant..

[12] Holick M.F. (2007). Vitamin D deficiency. N. Engl. J. Med..

[13] Bodnar L.M., Catov J.M., Simhan H.N., Holick M.F., Powers R.W., Roberts J.M. (2007). Maternal vitamin D deficiency increases the risk of preeclampsia. J. Clin. Endocrinol. Metab..

[14] Wellington K., Mulla Z.D. (2012). Seasonal trend in the occurrence of preeclampsia and eclampsia in Texas. Am. J. Hypertens..

[15] Jones G. (2008). Pharmacokinetics of vitamin D toxicity. Am. J. Clin. Nutr..

[16] Bikle D.D. (2010). Vitamin D: Newly discovered actions require reconsideration of physiologic requirements. Trends Endocrinol. Metab..

[17] Haussler M.R., Jurutka P.W., Mizwicki M., Norman A.W. (2011). Vitamin D receptor (VDR)-mediated actions of 1α,25(OH)_2_ vitamin D_3_: Genomic and non-genomic mechanisms. Best Pract. Res. Clin. Endocrinol. Metab..

[18] Carlberg C., Seuter S., Heikkinen S. (2012). The first genome-wide view of vitamin D receptor locations and their mechanistic implications. Anticancer Res..

[19] Woodham P.C., Brittain J.E., Baker A.M., Long D.L., Haeri S., Camargo C.A., Boggess K.A., Stuebe A.M. (2011). Midgestation maternal serum 25-hydroxyvitamin D level and soluble fms-like tyrosine kinase 1/placental growth factor ratio as predictors of severe preeclampsia. Hypertension.

[20] Wiseman H. (1993). Vitamin D is a membrane antioxidant. Ability to inhibit iron-dependent lipid peroxidation in liposomes compared to cholesterol, ergosterol and tamoxifen and relevance to anticancer action. FEBS Lett..

[21] Phillips J.K., Bernstein I.M., Mongeon J.A., Badger G.J. (2004). Seasonal variation in preeclampsia based on timing of conception. Obstet. Gynecol..

[22] Villar J., Abdel-Aleem H., Merialdi M., Mathai M., Ali M.M., Zavaleta N., Purwar M., Hofmeyr J., Nguyen T.N., Campodonico L. (2006). World Health Organization randomized trial of calcium supplementation among low calcium intake pregnant women. Am. J. Obstet. Gynecol..

[23] Mulligan M.L., Felton S.K., Riek A.E., Bernal-Mizrachi C. (2010). Implications of vitamin D deficiency in pregnancy and lactation. Am. J. Obstet. Gynecol..

[24] Agarwal A., Aponte-Mellado A., Premkumar B.J., Shaman A., Gupta S. (2012). The effects of oxidative stress on female reproduction: A review. Reprod. Biol. Endocrinol..

[25] Tabacova S., Little R.E., Balabaeva L., Pavlova S., Petrov I. (1994). Complications of pregnancy in relation to maternal lipid peroxides, glutathione, and exposure to metals. Reprod. Toxicol..

[26] Walsh S.W. (1998). Maternal-placental interactions of oxidative stress and antioxidants in preeclampsia. Semin. Reprod. Endocrinol..

[27] Wisdom S.J., Wilson R., McKillop J.H., Walker J.J. (1991). Antioxidant systems in normal pregnancy and in pregnancy-induced hypertension. Am. J. Obstet. Gynecol..

[28] Davidge S.T., Hubel C.A., Brayden R.D., Capeless E.C., McLaughlin M.K. (1992). Sera antioxidant activity in uncomplicated and preeclamptic pregnancies. Obstet. Gynecol..

[29] Morris J.M., Gopaul N.K., Endresen M.J., Knight M., Linton E.A., Dhir S., Anggard E.E., Redman C.W. (1998). Circulating markers of oxidative stress are raised in normal pregnancy and pre-eclampsia. Br. J. Obstet. Gynaecol..

[30] Strauss J.F., Martinez F., Kiriakidou M. (1996). Placental steroid hormone synthesis: Unique features and unanswered questions. Biol. Reprod..

[31] Tuckey R.C. (2005). Progesterone synthesis by the human placenta. Placenta.

[32] Klimek J., Wozniak M., Szymanska G., Zelewski L. (1998). Inhibitory effect of free radicals derived from organic hydroperoxide on progesterone synthesis in human term placental mitochondria. Free Radic. Biol. Med..

[33] Chwalisz K., Garfield R.E. (1994). Role of progesterone during pregnancy: Models of parturition and preeclampsia. Z. Geburtshilfe Perinatol..

[34] Slominski A.T., Zmijewski M.A., Semak I., Zbytek B., Pisarchik A., Li W., Zjawiony J., Tuckey R.C. (2014). Cytochromes P450 and skin cancer: Role of local endocrine pathways. Anticancer Agents Med. Chem..

[35] Slominski A.T., Kim T.K., Shehabi H.Z., Semak I., Tang E.K., Nguyen M.N., Benson H.A., Korik E., Janjetovic Z., Chen J. (2012). *In vivo* evidence for a novel pathway of vitamin D_3_ metabolism initiated by P450scc and modified by CYP27B1. FASEB J..

[36] Tuckey R.C., Li W., Zjawiony J.K., Zmijewski M.A., Nguyen M.N., Sweatman T., Miller D., Slominski A. (2008). Pathways and products for the metabolism of vitamin D_3_ by cytochrome P450scc. FEBS J..

[37] Diaz L., Sanchez I., Avila E., Halhali A., Vilchis F., Larrea F. (2000). Identification of a 25-hydroxyvitamin D_3_ 1α-hydroxylase gene transcription product in cultures of human syncytiotrophoblast cells. J. Clin. Endocrinol. Metab..

[38] Pospechova K., Rozehnal V., Stejskalova L., Vrzal R., Pospisilova N., Jamborova G., May K., Siegmund W., Dvorak Z., Nachtigal P. (2009). Expression and activity of vitamin D receptor in the human placenta and in choriocarcinoma BeWo and JEG-3 cell lines. Mol. Cell. Endocrinol..

[39] Anari M.R., Khan S., O’Brien P.J. (1996). The involvement of cytochrome P450 peroxidase in the metabolic bioactivation of cumene hydroperoxide by isolated rat hepatocytes. Chem. Res. Toxicol..

[40] Weiss R.H., Estabrook R.W. (1986). The mechanism of cumene hydroperoxide-dependent lipid peroxidation: The function of cytochrome P-450. Arch. Biochem. Biophys..

[41] Levin W., Lu A.Y., Jacobson M., Kuntzman R., Poyer J.L., McCay P.B. (1973). Lipid peroxidation and the degradation of cytochrome P-450 heme. Arch. Biochem. Biophys..

[42] Yao K., Falick A.M., Patel N., Correia M.A. (1993). Cumene hydroperoxide-mediated inactivation of cytochrome P450 2B1. Identification of an active site heme-modified peptide. J. Biol. Chem..

[43] Barr D.P., Martin M.V., Guengerich F.P., Mason R.P. (1996). Reaction of cytochrome P450 with cumene hydroperoxide: ESR spin-trapping evidence for the homolytic scission of the peroxide O–O bond by ferric cytochrome P450 1A2. Chem. Res. Toxicol..

[44] Rota C., Barr D.P., Martin M.V., Guengerich F.P., Tomasi A., Mason R.P. (1997). Detection of free radicals produced from the reaction of cytochrome P-450 with linoleic acid hydroperoxide. Biochem. J..

[45] Szymczyk G., Beltowski J., Marciniak A., Kotarski J. (2005). Serum isoprostanes levels in patients after abdominal hysterectomy. Rocz. Akad. Med. Bialymst..

[46] Scholl T.O., Leskiw M., Chen X., Sims M., Stein T.P. (2005). Oxidative stress, diet, and the etiology of preeclampsia. Am. J. Clin. Nutr..

[47] Tetteh P.W., Antwi-Boasiako C., Gyan B., Antwi D., Adzaku F., Obed S. (2013). Impaired renal function and increased urinary isoprostane excretion in Ghanaian women with pre-eclampsia. Res. Rep. Trop. Med..

[48] Barden A., Ritchie J., Walters B., Michael C., Rivera J., Mori T., Croft K., Beilin L. (2001). Study of plasma factors associated with neutrophil activation and lipid peroxidation in preeclampsia. Hypertension.

[49] McKinney E.T., Shouri R., Hunt R.S., Ahokas R.A., Sibai B.M. (2000). Plasma, urinary, and salivary 8-epi-prostaglandin f2α levels in normotensive and preeclamptic pregnancies. Am. J. Obstet. Gynecol..

[50] Ishihara O., Hayashi M., Osawa H., Kobayashi K., Takeda S., Vessby B., Basu S. (2004). Isoprostanes, prostaglandins and tocopherols in pre-eclampsia, normal pregnancy and non-pregnancy. Free Radic. Res..

[51] Basu S. (2004). Isoprostanes: Novel bioactive products of lipid peroxidation. Free Radic. Res..

[52] Basu S. (2010). Fatty acid oxidation and isoprostanes: Oxidative strain and oxidative stress. Prostaglandins Leukot. Essent. Fatty Acids.

[53] Fam S.S., Morrow J.D. (2003). The isoprostanes: Unique products of arachidonic acid oxidation—A review. Curr. Med. Chem..

[54] Barden A., Beilin L.J., Ritchie J., Croft K.D., Walters B.N., Michael C.A. (1996). Plasma and urinary 8-iso-prostane as an indicator of lipid peroxidation in pre-eclampsia and normal pregnancy. Clin. Sci..

[55] Regan C.L., Levine R.J., Baird D.D., Ewell M.G., Martz K.L., Sibai B.M., Rokach J., Lawson J.A., Fitzgerald G.A. (2001). No evidence for lipid peroxidation in severe preeclampsia. Am. J. Obstet. Gynecol..

[56] Kalpdev A., Saha S.C., Dhawan V. (2011). Vitamin C and E supplementation does not reduce the risk of superimposed PE in pregnancy. Hypertens. Pregnancy.

[57] Shin J.S., Choi M.Y., Longtine M.S., Nelson D.M. (2010). Vitamin D effects on pregnancy and the placenta. Placenta.

[58] Slominski A.T., Kim T.K., Chen J., Nguyen M.N., Li W., Yates C.R., Sweatman T., Janjetovic Z., Tuckey R.C. (2012). Cytochrome P450scc-dependent metabolism of 7-dehydrocholesterol in placenta and epidermal keratinocytes. Int. J. Biochem. Cell Biol..

[59] Urrutia R.P., Thorp J.M. (2012). Vitamin D in pregnancy: Current concepts. Curr. Opin. Obstet. Gynecol..

[60] Hollis B.W., Wagner C.L. (2011). Vitamin D requirements and supplementation during pregnancy. Curr. Opin. Endocrinol. Diabetes Obes..

[61] Bandeira F., Griz L., Dreyer P., Eufrazino C., Bandeira C., Freese E. (2006). Vitamin D deficiency: A global perspective. Arq Bras. Endocrinol. Metabol..

[62] Bodnar L.M., Simhan H.N., Powers R.W., Frank M.P., Cooperstein E., Roberts J.M. (2007). High prevalence of vitamin D insufficiency in black and white pregnant women residing in the northern United States and their neonates. J. Nutr..

[63] Holmes V.A., Barnes M.S., Alexander H.D., McFaul P., Wallace J.M. (2009). Vitamin D deficiency and insufficiency in pregnant women: A longitudinal study. Br. J. Nutr..

[64] O’Riordan M.N., Kiely M., Higgins J.R., Cashman K.D. (2008). Prevalence of suboptimal vitamin D status during pregnancy. Ir. Med. J..

[65] Kmiec P., Zmijewski M., Waszak P., Sworczak K., Lizakowska-Kmiec M. (2014). Vitamin D deficiency during winter months among an adult, predominantly urban, population in Northern Poland. Endokrynol. Pol..

[66] Kmiec P., Zmijewski M., Lizakowska-Kmiec M., Sworczak K. (2015). Widespread vitamin D deficiency among adults from northern Poland (54 degrees N) after months of low and high natural UVB radiation. Endokrynol. Pol..

[67] Pludowski P., Karczmarewicz E., Bayer M., Carter G., Chlebna-Sokol D., Czech-Kowalska J., Debski R., Decsi T., Dobrzanska A., Franek E. (2013). Practical guidelines for the supplementation of vitamin D and the treatment of deficits in Central Europe-recommended vitamin D intakes in the general population and groups at risk of vitamin D deficiency. Endokrynol. Pol..

[68] Bodnar L.M., Simhan H.N., Catov J.M., Roberts J.M., Platt R.W., Diesel J.C., Klebanoff M.A. (2014). Maternal vitamin D status and the risk of mild and severe preeclampsia. Epidemiology.

[69] Wang Y., Walsh S.W. (1998). Placental mitochondria as a source of oxidative stress in pre-eclampsia. Placenta.

[70] Walsh S.W., Wang Y. (1995). Trophoblast and placental villous core production of lipid peroxides, thromboxane, and prostacyclin in preeclampsia. J. Clin. Endocrinol. Metab..

[71] Walsh S.W. (2004). Eicosanoids in preeclampsia. Prostaglandins Leukot. Essent. Fatty Acids.

[72] Morikawa S., Kurauchi O., Tanaka M., Yoneda M., Uchida K., Itakura A., Furugori K., Mizutani S., Tomoda Y. (1997). Increased mitochondrial damage by lipid peroxidation in trophoblast cells of preeclamptic placentas. Biochem. Mol. Biol. Int..

[73] He G., Xu W., Chen Y., Liu X., Xi M. (2013). Abnormal apoptosis of trophoblastic cells is related to the up-regulation of CYP11A gene in placenta of preeclampsia patients. PLoS ONE.

[74] Moon J.Y., Moon M.H., Kim K.T., Jeong D.H., Kim Y.N., Chung B.C., Choi M.H. (2014). Cytochrome P450-mediated metabolic alterations in preeclampsia evaluated by quantitative steroid signatures. J. Steroid Biochem. Mol. Biol..

[75] Hallmann A., Klimek J., Masaoka M., Kaminski M., Kedzior J., Majczak A., Niemczyk E., Wozniak M., Trzonkowski P., Wakabayashi T. (2004). Partial characterization of human choriocarcinoma cell line JAR cells in regard to oxidative stress. Acta Biochim. Pol..

[76] Hallmann A., Milczarek R., Lipinski M., Kossowska E., Spodnik J.H., Wozniak M., Wakabayashi T., Klimek J. (2004). Fast perinuclear clustering of mitochondria in oxidatively stressed human choriocarcinoma cells. Folia Morphol..

[77] Ma R., Gu Y., Zhao S., Sun J., Groome L.J., Wang Y. (2012). Expressions of vitamin D metabolic components VDBP, CYP2R1, CYP27B1, CYP24A1, and VDR in placentas from normal and preeclamptic pregnancies. Am. J. Physiol. Endocrinol. Metab..

[78] Diaz L., Arranz C., Avila E., Halhali A., Vilchis F., Larrea F. (2002). Expression and activity of 25-hydroxyvitamin D-1α-hydroxylase are restricted in cultures of human syncytiotrophoblast cells from preeclamptic pregnancies. J. Clin. Endocrinol. Metab..

[79] Halhali A., Tovar A.R., Torres N., Bourges H., Garabedian M., Larrea F. (2000). Preeclampsia is associated with low circulating levels of insulin-like growth factor I and 1,25-dihydroxyvitamin D in maternal and umbilical cord compartments. J. Clin. Endocrinol. Metab..

[80] Baker A.M., Haeri S., Camargo C.A., Espinola J.A., Stuebe A.M. (2010). A nested case-control study of midgestation vitamin D deficiency and risk of severe preeclampsia. J. Clin. Endocrinol. Metab..

[81] Gutteridge J.M. (1986). Iron promoters of the Fenton reaction and lipid peroxidation can be released from haemoglobin by peroxides. FEBS Lett..

[82] Braughler J.M., Duncan L.A., Chase R.L. (1986). The involvement of iron in lipid peroxidation. Importance of ferric to ferrous ratios in initiation. J. Biol. Chem..

[83] Walsh S.W., Parisi V.M. (1986). The role of arachidonic acid metabolites in preeclampsia. Semin. Perinatol..

[84] Wang Y.P., Walsh S.W., Guo J.D., Zhang J.Y. (1991). The imbalance between thromboxane and prostacyclin in preeclampsia is associated with an imbalance between lipid peroxides and vitamin E in maternal blood. Am. J. Obstet. Gynecol..

[85] Warso M.A., Lands W.E. (1983). Lipid peroxidation in relation to prostacyclin and thromboxane physiology and pathophysiology. Br. Med. Bull..

[86] Huang C.H., Ren F.R., Shan G.Q., Qin H., Mao L., Zhu B.Z. (2015). Molecular mechanism of metal-independent decomposition of organic hydroperoxides by the halogenated quinoid carcinogens and the potential biological implications. Chem. Res. Toxicol..

[87] Abad C., Vargas F.R., Zoltan T., Proverbio T., Pinero S., Proverbio F., Marin R. (2015). Magnesium sulfate affords protection against oxidative damage during severe preeclampsia. Placenta.

[88] Acikgoz S., Bayar U.O., Can M., Guven B., Mungan G., Dogan S., Sumbuloglu V. (2013). Levels of oxidized LDL, estrogens, and progesterone in placenta tissues and serum paraoxonase activity in preeclampsia. Mediators Inflamm..

[89] Walsh S.W. (1988). Progesterone and estradiol production by normal and preeclamptic placentas. Obstet. Gynecol..

[90] Walsh S.W. (1985). Preeclampsia: An imbalance in placental prostacyclin and thromboxane production. Am. J. Obstet. Gynecol..

[91] Walsh S.W., Coulter S. (1989). Increased placental progesterone may cause decreased placental prostacyclin production in preeclampsia. Am. J. Obstet. Gynecol..

[92] Fitzgerald D.J., Rocki W., Murray R., Mayo G., Fitzgerald G.A. (1990). Thromboxane A2 synthesis in pregnancy-induced hypertension. Lancet.

[93] Hogg K., Blair J.D., McFadden D.E., von D.P., Robinson W.P. (2013). Early onset pre-eclampsia is associated with altered DNA methylation of cortisol-signalling and steroidogenic genes in the placenta. PLoS ONE.

[94] Tamimi R., Lagiou P., Vatten L.J., Mucci L., Trichopoulos D., Hellerstein S., Ekbom A., Adami H.O., Hsieh C.C. (2003). Pregnancy hormones, pre-eclampsia, and implications for breast cancer risk in the offspring. Cancer Epidemiol. Biomark. Prev..

[95] Beaudoin C., Blomquist C.H., Bonenfant M., Tremblay Y. (1997). Expression of the genes for 3 β-hydroxysteroid dehydrogenase type 1 and cytochrome P450scc during syncytium formation by human placental cytotrophoblast cells in culture and the regulation by progesterone and estradiol. J. Endocrinol..

[96] Risberg A., Olsson K., Lyrenas S., Sjoquist M. (2009). Plasma vasopressin, oxytocin, estradiol, and progesterone related to water and sodium excretion in normal pregnancy and gestational hypertension. Acta Obstet. Gynecol. Scand..

[97] Meher S., Duley L. (2006). Progesterone for preventing pre-eclampsia and its complications. Cochrane. Database. Syst. Rev..

[98] Al E.S., Hammoudeh M. (2013). Vitamin D study in pregnant women and their babies. Qatar Med. J..

[99] Slominski A.T., Li W., Kim T.K., Semak I., Wang J., Zjawiony J.K., Tuckey R.C. (2014). Novel activities of CYP11A1 and their potential physiological significance. J. Steroid Biochem. Mol. Biol..

[100] Slominski A., Semak I., Zjawiony J., Wortsman J., Li W., Szczesniewski A., Tuckey R.C. (2005). The cytochrome P450scc system opens an alternate pathway of vitamin D3 metabolism. FEBS J..

[101] Slominski A.T., Kim T.K., Li W., Yi A.K., Postlethwaite A., Tuckey R.C. (2014). The role of CYP11A1 in the production of vitamin D metabolites and their role in the regulation of epidermal functions. J. Steroid Biochem. Mol. Biol..

[102] Tuckey R.C., Nguyen M.N., Slominski A. (2008). Kinetics of vitamin D3 metabolism by cytochrome P450scc (CYP11A1) in phospholipid vesicles and cyclodextrin. Int. J. Biochem. Cell Biol..

[103] Haugen M., Brantsaeter A.L., Trogstad L., Alexander J., Roth C., Magnus P., Meltzer H.M. (2009). Vitamin D supplementation and reduced risk of preeclampsia in nulliparous women. Epidemiology.

[104] Hathcock J.N., Shao A., Vieth R., Heaney R. (2007). Risk assessment for vitamin D. Am. J. Clin. Nutr..

[105] Heaney R.P. (2008). Vitamin D: Criteria for safety and efficacy. Nutr. Rev..

[106] Aloia J.F., Patel M., Dimaano R., Li-Ng M., Talwar S.A., Mikhail M., Pollack S., Yeh J.K. (2008). Vitamin D intake to attain a desired serum 25-hydroxyvitamin D concentration. Am. J. Clin. Nutr..

[107] Hollis B.W., Johnson D., Hulsey T.C., Ebeling M., Wagner C.L. (2011). Vitamin D supplementation during pregnancy: Double-blind, randomized clinical trial of safety and effectiveness. J. Bone Miner. Res..

[108] Roth D.E., Al M.A., Raqib R., Akhtar E., Perumal N., Pezzack B., Baqui A.H. (2013). Randomized placebo-controlled trial of high-dose prenatal third-trimester vitamin D3 supplementation in Bangladesh: The AViDD trial. Nutr. J..

[109] Sablok A., Batra A., Thariani K., Batra A., Bharti R., Aggarwal A.R., Kabi B.C., Chellani H. (2015). Supplementation of vitamin D in pregnancy and its correlation with feto-maternal outcome. Clin. Endocrinol..

[110] Wang J., Slominski A., Tuckey R.C., Janjetovic Z., Kulkarni A., Chen J., Postlethwaite A.E., Miller D., Li W. (2012). 20-hydroxyvitamin D_3_ inhibits proliferation of cancer cells with high efficacy while being non-toxic. Anticancer Res..

[111] Slominski A.T., Janjetovic Z., Fuller B.E., Zmijewski M.A., Tuckey R.C., Nguyen M.N., Sweatman T., Li W., Zjawiony J., Miller D. (2010). Products of vitamin D3 or 7-dehydrocholesterol metabolism by cytochrome P450scc show anti-leukemia effects, having low or absent calcemic activity. PLoS ONE.

[112] Slominski A., Janjetovic Z., Tuckey R.C., Nguyen M.N., Bhattacharya K.G., Wang J., Li W., Jiao Y., Gu W., Brown M. (2013). 20S-hydroxyvitamin D3, noncalcemic product of CYP11A1 action on vitamin D3, exhibits potent antifibrogenic activity *in vivo*. J. Clin. Endocrinol. Metab..

[113] Janjetovic Z., Zmijewski M.A., Tuckey R.C., DeLeon D.A., Nguyen M.N., Pfeffer L.M., Slominski A.T. (2009). 20-Hydroxycholecalciferol, product of vitamin D3 hydroxylation by P450scc, decreases NF-κB activity by increasing IkappaB α levels in human keratinocytes. PLoS ONE.

[114] Janjetovic Z., Tuckey R.C., Nguyen M.N., Thorpe E.M., Slominski A.T. (2010). 20,23-dihydroxyvitamin D3, novel P450scc product, stimulates differentiation and inhibits proliferation and NF-kappaB activity in human keratinocytes. J. Cell Physiol..

[115] Chen J., Wang J., Kim T.K., Tieu E.W., Tang E.K., Lin Z., Kovacic D., Miller D.D., Postlethwaite A., Tuckey R.C. (2014). Novel vitamin D analogs as potential therapeutics: Metabolism, toxicity profiling, and antiproliferative activity. Anticancer Res..

[116] Castejon O.C.S. (2011). Mitochondrial dysfunction and apoptosis in trophoblast cells during preeclampsia: And ultrastural study. Electron. J. Biomed..

[117] Sobek J., Martschke R., Fischer H. (2001). Entropy control of the cross-reaction between carbon-centered and nitroxide radicals. J. Am. Chem. Soc..

[118] Damiani E., Kalinska B., Canapa A., Canestrari S., Wozniak M., Olmo E., Greci L. (2000). The effects of nitroxide radicals on oxidative DNA damage. Free Radic. Biol. Med..

[119] Chateauneuf J., Lusztyk J., Ingold K.U. (1988). Absolute rate constants for the reactions of some carbon-centered radicals with 2,2,6,6-tetramethyl-1-piperidinoxyl. J. Org. Chem..

[120] Mitchell J.B., Samuni A., Krishna M.C., DeGraff W.G., Ahn M.S., Samuni U., Russo A. (1990). Biologically active metal-independent superoxide dismutase mimics. Biochemistry.

[121] McCance D.R., Holmes V.A., Maresh M.J., Patterson C.C., Walker J.D., Pearson D.W., Young I.S. (2010). Vitamins C and E for prevention of pre-eclampsia in women with type 1 diabetes (DAPIT): A randomised placebo-controlled trial. Lancet.

[122] Klimek J. (1988). The involvement of superoxide and iron ions in the NADPH-dependent lipid peroxidation in human placental mitochondria. Biochim. Biophys. Acta.

[123] Omura T., Sato R. (1964). The carbon monoxide-binding pigment of liver microsomes. I. Evidence for its hemoprotein nature. J. Biol. Chem..

[124] Thompson E.A., Siiteri P.K. (1974). The involvement of human placental microsomal cytochrome P-450 in aromatization. J. Biol. Chem..

